# Women Born Preterm or with Inappropriate Weight for Gestational Age Are at Risk of Subsequent Gestational Diabetes and Pre-Eclampsia

**DOI:** 10.1371/journal.pone.0034001

**Published:** 2012-03-29

**Authors:** Rasmus á Rogvi, Julie Lyng Forman, Peter Damm, Gorm Greisen

**Affiliations:** 1 Department of Neonatology, Copenhagen University Hospital, Copenhagen, Denmark; 2 Department of Biostatistics, Copenhagen University, Copenhagen, Denmark; 3 Department of Obstetrics, Center for Pregnant Women with Diabetes, Copenhagen University Hospital, Copenhagen, Denmark; Indiana University, United States of America

## Abstract

**Introduction:**

Low birthweight, which can be caused by inappropriate intrauterine growth or prematurity, is associated with development of gestational diabetes mellitus (GDM) as well as pre-eclampsia later in life, but the relative effects of prematurity and inappropriate intrauterine growth remain uncertain.

**Methods:**

Through nation-wide registries we identified all Danish mothers in the years 1989–2007. Two separate cohorts consisting mothers born 1974–1977 (n = 84219) and 1978–1981 (n = 32376) were studied, due to different methods of registering birthweight and gestational age in the two periods. Data was linked with information on GDM, pre-eclampsia and education.

**Results:**

In a multivariate logistic regression model the odds of developing GDM was increased by 5–7% for each week the mother was born before term (p = 0.018 for 1974–1977, p = 0.048 for 1978–1981), while the odds were increased by 13–17% for each standard deviation (SD) reduction in birthweight for gestational age for those who were small or normal for gestational age (p<0.0001 and p = 0.035) and increased by 118–122% for each SD increase above the normal range (p<0.0001 and p = 0.024). The odds of pre-eclampsia was increased by 3–5% for each week the mother was born before term (p = 0.064 and p = 0.04), while the odds were increased 11–12% for each SD reduction in birthweight for gestational age (p<0.0001 and p = 0.0002).

**Conclusion:**

In this cohort of young Danish mothers, being born premature or with increasingly low birthweight for gestational age was associated with an increased risk of GDM and pre-eclampsia in adulthood, while increasingly high birthweight for gestational age was associated with an increased risk of GDM and a decreased risk of pre-eclampsia. Inappropriate weight for gestational age was a more important risk factor than prematurity.

## Introduction

Low birthweight (LBW) is known to be an important risk factor for infant mortality and morbidity and development of disease in adult life [1], [2]. The hypothesis of the developmental origins of adult disease, also known as the Barker hypothesis, states that suboptimal conditions in intrauterine life increases the risk of disease in adult life [3], [4]. LBW has, among other risk factors, been shown to be associated with development of GDM, pre-eclampsia as well as T2D later in life [5]–[9]. LBW is caused by prematurity, poor fetal growth or both, but the relative importance of these factors is uncertain.

Children born preterm, with a normal fetal growth, are showing increased insulin resistance in both childhood and early adult life compared to children born at term, and maternal insulin resistance has been shown to be a risk factor for preeclampsia [10]–[12]. Furthermore both preterm birth and poor fetal growth are associated with an increased risk of T2D in adult life [13]. The risk of GDM has been shown to be associated with being born small for gestational age (SGA), large for gestational age (LGA) as well as preterm birth in a univariate analysis, but the factors were not included in a multivariate analysis [14]. The risk of pre-eclampsia is increased in mothers born SGA, but we could not identify studies on the risk for women born preterm, LGA or studies that combined prematurity and inappropriate growth in the analysis [15].

GDM is associated with development of T2D later in life, and pre-eclampsia is associated with T2D as well as adverse cardiovascular events but GDM and pre-eclampsia usually appear several years before these events [16]–[19]. As such, both are also important as early markers of subsequent metabolic and cardiovascular disease in adulthood.

With this study we wanted to test our hypothesis that prematurity and birthweight for gestational age increases the risk of developing GDM and pre-eclampsia, by including both factors in a multivariate analysis on a nationwide register-based cohort.

## Methods

Data were extracted in December 2008 from the The Danish Medical Birth Registry and The National Patient Registry held by The National Board of Health in Denmark and The Educational Registry held by Statistics Denmark, for a study on the risk of psychiatric disorders in children born preterm [20]. Details on the extraction and creation of the cohort can be found in this publication. Core characteristics of the two cohorts can be seen in Table 1. All persons born in or immigrated to Denmark are assigned a Central Personal Registry (CPR) number and data from the registries were linked through these numbers [21].

**Table 1:** Core characteristics of the two cohorts.

	1974–1977	1978–1981
Number of mothers	84219	32376
Maternal BW□	3301 (522)	3313 (531)
Maternal GA*	277.2 (9.3)	278.1 (11.2)
Maternal SDS†	−0.35 (1.08)	−0.37 (1.09)
Maternal age•	26.6 (3.5)	24.7 (2.82)
% with highest level of education	35.27%	22.85%
% with GDM	1.07%	0.81%
% with pre-eclampsia	2.1%	1.7%
Child BW□	3392 (681)	3399 (663)
Child GA*	278.2 (17.2)	278.1 (18.6)

□ Mean birthweight in grams (standard deviation).

* Mean gestational age in days (standard deviation).

† Mean birthweight by gestational age z-score (standard deviation).

• Mean maternal age in years (standard deviation).

The Danish Medical Birth Registry provided data on birthweight, gestational age, sex and CPR number on all people born in Denmark from 1974–2007. The National Patient Registry provided data on people diagnosed with GDM and pre-eclampsia in the same period. The Educational Registry provided data on the highest completed education. The data in all registries used are available for anyone to extract when approved by the Danish Data Protection Agency, and the study is thus reproducible.

Due to different methods of registering birthweight and gestational age in the periods 1974–1977 and 1978–1981, two different cohorts were created for this study [21]. The first cohort consisted of women born between 1974–1977 with a registered birthweight and gestational age in the Danish Medical Birth Registry (n = 130638), but only women with a registered childbirth in the years 1989–2007 were included (n = 84219). The second cohort consisted of women born between 1978–1981 with a registered birthweight and gestational age in the Danish Medical Birth Registry (n = 86715), but only women with a registered childbirth in the years 1989–2007 were included (n = 32376). Birthweight standard deviation z-score (SDS) was calculated by the use of Marsal's charts of normal intrauterine growth [22].

Data on pre-eclampsia and GDM were extracted from The National Patient Registry. From 1978–1993 it was coded using the ICD-8 classification, from 1994 onwards using the ICD-10 classification. We selected all women with a diagnosis of pre-eclampsia and/or eclampsia (ICD-8: 637.03, 637.04, 637.09 and 637.19, ICD-10: O14 and O15) or GDM (ICD-8: 634.74, ICD-10: O24.4). A woman was identified as a positive case if she was registered as having GDM or pre-eclampsia in at least one pregnancy.

Data on highest completed education was extracted as a distinct code for the highest achieved level of education the woman had completed by December 2007 and was recoded into 4 levels: 1) completed primary school, 2) completed high-school or 2–3 years of skilled work education after primary school, 3) completed a short higher education after high-school (2–3 years length) and 4) completed a long higher education after high-school (5 years length or more).

### Ethics

The data collection for the study was approved by the Danish Data Protection Agency. The CPR numbers were encrypted and thus anonymised for the researchers. Under Danish legislation it is not necessary to apply for approval by The Danish National Committee on Biomedical Research Ethics for database studies, as long as the study is approved by the Danish Data Protection Agency. Nor is it necessary to get written consent from individuals for database studies.

### Statistical analysis

The frequency of GDM and pre-eclampsia was calculated for each cohort, as well as for intervals of gestational age and SDS. To distinguish the effects of inappropriate fetal growth and gestational age we applied a multivariate logistic regression model including gestational age (completed days of gestation) and SDS (calculated from the normal fetal growth reference) as continuous predictors, and with further adjustment for maternal age at giving birth (continuous predictor) and highest completed education (four categories as described in the above) [22]. For all analyses a p-value of <0.05 was considered statistically significant. In order to test the U-shaped distribution of the frequency of GDM and pre-eclampsia by SDS, the effect of SDS was initially modeled by a linear spline with breakpoints at −3, −2, −1, 0, 1, 2, and 3 SD representing potential changes in the rate of increase/decrease of risk with SDS. Subsequently all breakpoints that did not reach the level of significance were removed in a step-wise backwards elimination procedure. The odds ratio (OR) for GDM and pre-eclampsia for an incremental change in SDS and gestational age was finally estimated in the reduced model. The OR for gestational age was computed as the relative increase in odds associated with a decrease in gestational age by one week, while the OR for SDS was computed as the relative increase in odds associated with a 1 SD increase or decrease in the SDS. Finally we tested for interactions between the SDS and maternal age and maternal educational level. Similar analyses were applied to both of the cohorts. The statistical analyses were all carried out by means of SAS 9.2 (SAS Institute, Inc, Cary, NC).

## Results

The first cohort consisted of 84219 mothers with a mean age at birth of 26.6 years (SD±3.5 years) (Table 1). In this cohort 2.2% of the pregnancies were complicated by pre-eclampsia (n = 1841) and 1.1% by GDM (n = 898).

The second cohort consisted of 32376 mothers with a mean age at birth of 24.7 years (SD±2.8 years). In this cohort 1.8% of the pregnancies were complicated by pre-eclampsia (n = 590) and 0.8% by GDM (n = 263).

**Figure 1:**
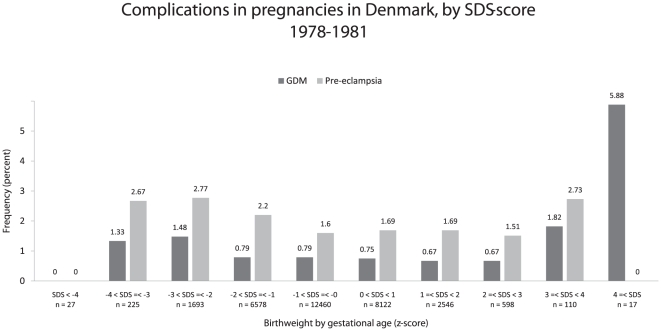
Frequency of GDM and pre-eclampsia by SDS, 1978–1981. The distribution of GDM shows a U-shaped pattern with an increased frequency for mothers born SGA and LGA, while the distribution of pre-eclampsia shows an approximate monotonous relationship, with increasing frequency of pre-eclampsia associated with smallness for gestational age.

**Figure 2:**
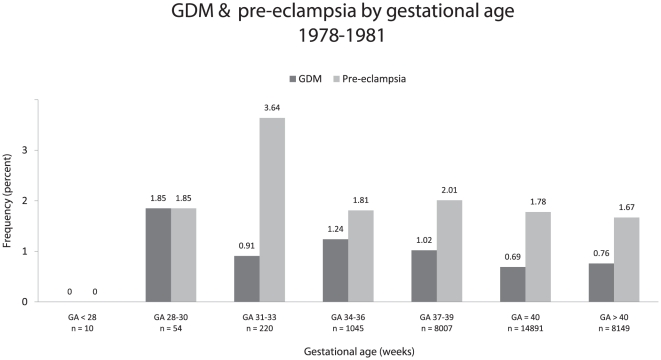
Frequency of GDM and pre-eclampsia by gestational age, 1978–1981. The distribution of both GDM and pre-eclampsia shows an approximate monotonous relationship, with increasing frequencies associated with preterm birth.

The distribution of GDM/pre-eclampsia by SDS can be seen in Figure 1. Due to the U-shaped distribution, SDS was modeled by a linear spline in the multivariate analysis as described in the statistical analysis section. The distribution of GDM and pre-eclampsia by gestational age can be seen in Figure 2. Due to the approximate monotonic relationship between gestational age and the risk of GDM/pre-eclampsia it was modeled as a simple linear effect in the multivariate logistic regression model.

After removing the insignificant breakpoints in the regression model, we ended up with a single breakpoint in the model for GDM with minimum risk for SDS = 2, while for pre-eclampsia no breakpoints, i.e. no significant deviations from linearity, were found (Table 2).

**Table 2:** Variables in the multivariate model.

	1974–1977		1978–1981	
GDM				
	p-value	OR	p-value	OR
GA*	0.018	1.05 [1.01–1.10]	0.048	1.07 [1.00–1.14]
SDS≤2†	<.0001	1.17 [1.10–1.25]	0.035	1.13 [1.01–1.27]
SDS>2‡	<.0001	2.22 [1.49–3.31]	0.024	2.18 [1.11–4.28]
Maternal Education•	<.0001	2.10 [1.71–2.58]	0.0007	2.21 [1.45–3.36]
Maternal Age□	0.25	1.01 [0.99–1.03]	0.038	1.05 [1.00–1.11]

Pre-eclampsia				
	p-value	OR	p-value	OR
GA*	0.064	1.03 [1.00–1.06]	0.04	1.05 [1.00–1.10]
SDS†	<.0001	1.11 [1.06–1.16]	0.002	1.12 [1.04–1.24]
Maternal Education•	0.12	0.94 [0.81–1.10]	0.13	1.03 [0.77–1.38]
Maternal Age□	<.0001	0.95 [0.94–0.96]	<.0001	0.93 [0.90–0.96]

* Gestational age (OR for a reduction of 1 week).

† Birthweight by gestational age z-score (OR for a reduction of 1 standard deviation).

‡ Birthweight by gestational age z-score, values >2 (OR for an increase of 1 standard deviation).

• Maternal level of education (OR for lowest vs. highest educational level).

□ Maternal age at birth (OR for an increase of 1 year).

For GDM the multivariate model showed an increased risk with low gestational age and a U-shaped distribution for maternal SDS, with the highest risk for mothers born with increasingly low birth for gestational age or increasingly high birthweight by gestational age. For pre-eclampsia the model showed an increased risk with low gestational age and a linear association with SDS. The model was corrected for maternal age and education, and the confounding factors were distributed as expected, with a significant effect of maternal educational level on GDM (higher educational level leads to lower risk of GDM) and a significant effect of maternal age on pre-eclampsia (low maternal age leads to higher risk of pre-eclampsia). No significant interactions between SDS and maternal age or educational level were found.

The risk of developing GDM increased significantly with decreasing gestational age (p = 0.018 for 1974–1977, p = 0.048 for 1978–1981), with decreasing SDS (p<0.0001 and p = 0.035) for SDS values less than 2, as well as with increasing SDS for values greater than 2 (p<0.0001 and p = 0.024) (Table 2). The risk of developing pre-eclampsia was significantly associated with low gestational age (p = 0.064 and p = 0.04) and decreasing SDS (p<0.0001 and p = 0.0002).

In both analyses we corrected for maternal age and educational level. GDM was significantly associated with lower maternal educational level, but not with maternal age, while pre-eclampsia was associated with lower maternal age but not with educational level (Table 2). No significant interactions between SDS and maternal age or maternal level of education were found.

For each week the mothers gestational age was shorter there were 5% [95% confidence interval 1–10%] increased odds of GDM for the first cohort, and 7% [0.1–14%] for the second cohort (Table 2). A reduction in SDS of 1 SD for values lower or equal to 2 SD increased the odds of GDM by 17% [10–25%] for the first cohort and 13% [1–27%] for the second cohort. An increase in SDS of 1 SD for values over 2 SD increased the odds by 122% [49–231%] and 118% [11–328%].

For each week the mothers gestational age was shorter there were 3% [−0.2–6.4%] increased odds of pre-eclampsia for the first cohort, and 5% [0.2–10%] for the second. A reduction in SDS of 1 SD increased the odds of pre-eclampsia by 11% [6–16%] for the first cohort and 12% [4–24%] for the second cohort.

**Figure 3:**
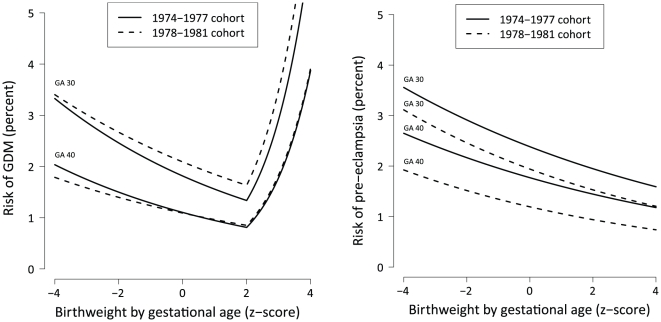
Modeled risk of GDM and preeclampsia by SDS. Risk curves given different gestational ages, here exemplified for a woman with a middle-long education giving birth at age 30. GA = gestational age in weeks.

Figure 3 exemplifies the estimated risk profile for a women aged 30 with a middle-long education, given different gestational ages and birthweight by gestational age.

## Discussion

Our results show that the risks of developing GDM and pre-eclampsia are both positively associated with maternal prematurity as well as smallness for gestational age when she was born herself. Furthermore largeness for gestational age is associated with an increased risk of GDM and a decreased risk of pre-eclampsia.

Our findings show general agreement between the two cohorts, with a higher level of significance in the first cohort, which is expected given its larger size and longer follow-up period. The overall agreement between the two cohorts shows that our findings are likely to be robust.

This study adds to the current knowledge by showing that prematurity, smallness for gestational age and largeness for gestational are independent and coexisting risk factors for developing GDM. It also shows that prematurity and smallness for gestational age are risk factors for developing pre-eclampsia, while the risk is not significantly increased for mothers born LGA.

One of the strengths of our study is the sample size, with over 115000 intergenerational births linked through nationwide registers. As with other database studies the loss to follow-up is minimal and there is no risk of recall bias.

The incidence of GDM of 0.8–1.1% was lower than the value found in another study of 2.4%. This might partly be explained by the relatively low maternal age in our cohorts (range 14–34 years, mean 24.7 and 26.6 years, mean age of primiparous women in Denmark 29.1 years) which also may explain the different incidences in the two cohorts. In addition not all women with GDM will get the diagnosis and not all women with the diagnosis will be registered in The National Patient Registry. A Swedish study using nationwide registries found an incidence of GDM of 0.9% [23]. The Patient Registry has not been validated for GDM, but for T2D the sensitivity has been found to be 64% with a positive predictive value of 97% [24]. If the same is the case for GDM this also explains a part of the lower-than-expected incidence, while the women identified as having GDM in our study are most likely true positive.

It is unknown whether the National Health Registry is influenced by selection bias in the GDM diagnoses, with women with higher socioeconomic status being more likely to get a GDM diagnosis. As the Danish National Health Service provides free antenatal care, diagnosis and treatment for all citizens, we would expect a selection bias to be modest. Given the increased risk of GDM with lower educational status, we would expect such a bias to influence our results towards the null.

The incidence of pre-eclampsia of 1.8–2.2% is also a bit lower than expected. Studies using The National Health Registry or The Danish Birth Cohort both find an incidence of 3% [25], [26]. Again, the slightly smaller incidence might be explained by the young maternal age in the cohort, though the risk of pre-eclampsia falls with maternal age. The validity of pre-eclampsia and hypertensive diseases in pregnancy in The National Patient Registry has been validated in three studies, with a sensitivity ranging from 65–75% and a positive predictive value ranging from 70–75% [27]–[29].

Both GDM and pre-eclampsia are characterized by insulin resistance, and pre-eclampsia develops more frequently in pregnancies complicated by diabetes [30]–[32]. Furthermore increasing maternal age and BMI are well known risk factors for GDM and pre-eclampsia and BMI increases with advancing age in women [33], [34]. Our cohorts were drawn from the very first years of the Danish Medical Birth Registry. Since the women in our study were younger than average for giving birth in Denmark (the population average is around 30 years) it is possible that the ORs may become larger - or smaller - when data from women who give birth later in life can be included [35].

A weakness of our study is the need to split the study into two study cohorts. This reduces the power of our statistical analyses, but due to the different way of registering birthweight and gestational age we used this conservative way of analysis, depending on qualitative addition of the results from the two cohorts. In the earlier cohort, birthweight as well as gestational age was coded in intervals, giving rise to some miscomputation of SGA [21]. It is therefore reassuring that the effect of birthweight by gestational age was consistent between the two cohorts.

In the years 1978–1980, 13–30% of women born in Denmark are missing information on gestational age due to a change in how the data was reported to Danish Medical Birth Registry [20], [21]. The mean birthweight of the persons without data on gestational age are comparable to those with this data, and therefore the individuals with missing data are expected to be a random sample of the total birth cohort [21]. Therefore it is expected that the effects of prematurity found in our study are valid.

Both maternal BMI and lower socio-economic class have been identified as risk factors for GDM and pre-eclampsia, but it was not possible to correct directly for these factors in our study [36]–[38]. As the level of education is a good surrogate marker for socioeconomic class, the correction for educational levels comprises a satisfying correction for socioeconomic class. We found a significant association between lower maternal educational level and risk of GDM as well as lower maternal age and pre-eclampsia, which was as expected given the demographic of the cohorts [14], [38].

One previous study found a significantly increased risk of pre-eclampsia in daughters of mothers who also had pre-eclampsia, independently of the risk of pre-eclampsia by being born SGA [15]. In our study it was not possible to correct for grand-maternal pre-eclampsia, and we do not know if it would influence on our results.

Many risk factors for pre-eclampsia and gestational diabetes are recognised, several of which we were not able to correct for [38]. We cannot exclude that these risk factors could be distributed unevenly among, for example, women born SGA compared to women born with an appropriate birth weight. As the birth weight and gestational age, however, precede these risk factors, the question is whether they truly are individual risk factors or rather are mediators of the metabolic programming induced by prematurity/inappropriate fetal growth.

The clinical significance of our study is primarily to help identify the pregnancies that are at risk of being complicated. As the risk profiles for SGA, LGA and prematurity are independent and co-existing, the greatest risk is for women born in the extremes – especially the women born premature as well as SGA or the women born very large for gestational age.

Furthermore the findings in our study are consistent with the Barker hypothesis by showing that a woman's risk of pregnancy complications is dependent on her own intrauterine conditions.

In conclusion, in this large cohort of young Danish mothers, prematurity as well as low birthweight for gestational age were significantly associated with increased risks of GDM and pre-eclampsia while high birthweight for gestational age was associated with an increased risk of GDM and a decreased risk of pre-eclampsia.

The odds of GDM were increased by approximately 5–7% for each week the mother was born before term, while the odds were increased by 13–17% for each SD reduction in birthweight for gestational age for those who were small or normal for gestational age and increased by 118–122% for each SD increase above the normal range.

The odds of pre-eclampsia were increased by 3–5% for each week the mother was born before term, while the odds were increased 11–12% for each SD reduction in birthweight for gestational age for small, normal, and large for gestational age alike.

This means that inappropriate birthweight for gestational age is more important than prematurity for the prevalence of GDM and pre-eclampsia at the population level and even for those born extremely preterm, the odds are raised by less than a factor of two.
